# Sudden cardiac death and pump failure death prediction in chronic heart failure by combining ECG and clinical markers in an integrated risk model

**DOI:** 10.1371/journal.pone.0186152

**Published:** 2017-10-11

**Authors:** Julia Ramírez, Michele Orini, Ana Mincholé, Violeta Monasterio, Iwona Cygankiewicz, Antonio Bayés de Luna, Juan Pablo Martínez, Pablo Laguna, Esther Pueyo

**Affiliations:** 1 Clinical Pharmacology Department, William Harvey Research Institute, John Vane Science Centre, Queen Mary University of London, Charterhouse Square, London, United Kingdom; 2 Institute of Cardiovascular Science, University College London, London, United Kingdom; 3 Barts Heart Centre, St Bartholomeus Hospital, London, United Kingdom; 4 Department of Computer Science, University of Oxford, Oxford, United Kingdom; 5 Universidad San Jorge, Campus Universitario, Villanueva de Gállego, Spain; 6 Department of Electrocardiology, Medical University of Lodz, Sterling Regional Center for Heart Diseases, Lodz, Poland; 7 Catalan Institute of Cardiovascular Sciences, Santa Creu I Sant Pau Hospital, Barcelona, Spain; 8 Biomedical Signal Interpretation and Computational Simulation (BSICoS) group, Aragón Institute of Engineering Research, IIS Aragón, University of Zaragoza, Zaragoza, Spain; 9 Biomedical Research Networking Center in Bioengineering, Biomaterials and Nanomedicine (CIBER-BBN), Zaragoza, Spain; Scuola Superiore Sant'Anna, ITALY

## Abstract

**Background:**

Sudden cardiac death (SCD) and pump failure death (PFD) are common endpoints in chronic heart failure (CHF) patients, but prevention strategies are different. Currently used tools to specifically predict these endpoints are limited. We developed risk models to specifically assess SCD and PFD risk in CHF by combining ECG markers and clinical variables.

**Methods:**

The relation of clinical and ECG markers with SCD and PFD risk was assessed in 597 patients enrolled in the MUSIC (MUerte Súbita en Insuficiencia Cardiaca) study. ECG indices included: turbulence slope (TS), reflecting autonomic dysfunction; T-wave alternans (TWA), reflecting ventricular repolarization instability; and T-peak-to-end restitution (Δ*α*^*Tpe*^) and T-wave morphology restitution (TMR), both reflecting changes in dispersion of repolarization due to heart rate changes. Standard clinical indices were also included.

**Results:**

The indices with the greatest SCD prognostic impact were gender, New York Heart Association (NYHA) class, left ventricular ejection fraction, TWA, Δ*α*^*Tpe*^ and TMR. For PFD, the indices were diabetes, NYHA class, Δ*α*^*Tpe*^ and TS. Using a model with only clinical variables, the hazard ratios (HRs) for SCD and PFD for patients in the high-risk group (fifth quintile of risk score) with respect to patients in the low-risk group (first and second quintiles of risk score) were both greater than 4. HRs for SCD and PFD increased to 9 and 11 when using a model including only ECG markers, and to 14 and 13, when combining clinical and ECG markers.

**Conclusion:**

The inclusion of ECG markers capturing complementary pro-arrhythmic and pump failure mechanisms into risk models based only on standard clinical variables substantially improves prediction of SCD and PFD in CHF patients.

## Introduction

Sudden cardiac death (SCD) and pump failure death (PFD) are both common endpoints in patients with chronic heart failure (CHF) [1]. Prevention of these two different modes of death requires different treatment, including implantable cardioverter defibrillators (ICDs) to reduce SCD mortality [2] and cardiac resynchronization therapy to decrease PFD rate [3]. Effective techniques for risk stratification, able to specifically target functional (PFD) or arrhythmic (SCD) risk are needed.

Chronic heart failure is a complex clinical syndrome that can result from a number of functional or structural cardiac disorders, impairing the ventricle’s ability to fill with or eject blood [4]. A common finding in CHF patients is chronic sympathetic over-activity [5], a risk factor for both SCD and PFD [6, 7]. Initial sustained sympathetic activation increases the vulnerability to ventricular arrhythmias by enhancing the spatio-temporal heterogeneity of repolarization [8] and the ventricular response to heart rate changes [9]. Enduring sympathetic activation facilitates withdrawal of vagal activity [10] and a weakened ventricular response [11]. Considering the interaction of multiple factors in SCD and PFD, the combination of indices reflecting complementary mechanisms rather than the use of individual markers may improve SCD and PFD risk stratification.

Risk models based on clinical variables have been proposed for risk stratifying CHF patients at risk of SCD or PFD [12, 13, 14] and can be utilized as a first step to identify SCD or PFD risk subgroups [15]. In this study, we hypothesized that integrated models combining standard clinical variables with ECG markers assessing autonomic nervous system (ANS) imbalance and cardiac electrophysiological abnormalities would improve both SCD and PFD risk stratification, thus providing a tool to better discriminate between SCD and PFD risk. On top of the analysis on the general study population, separate analyses were performed in subpopulations of patients with reduced left ventricular ejection fraction (LVEF)≤35% (HFrEF group) and with preserved LVEF>35% (HFpEF group).

## Methods

### Study population

The original study population consisted of 992 consecutive patients with symptomatic CHF corresponding to NYHA classes II and III enrolled in the MUSIC (MUerte Súbita en Insuficiencia Cardiaca) study, a prospective, multicenter study designed to assess risk predictors for cardiovascular mortality in ambulatory CHF patients [13]. Patients were enrolled from the specialized CHF clinics of eight University Hospitals between April 2003 and December 2004. A two- (3%) or three-lead (97%) 24-h Holter ECG sampled at 200 Hz was recorded in each patient at enrolment using ELA Medical equipment (Sorin Group, Paris, France). The original cohort included patients in atrial fibrillation, in sinus rhythm, in flutter and in pacemaker rhythm. In this work, only data from patients in sinus rhythm (n = 651) were analyzed. The MUSIC study included patients with both reduced and preserved LVEF. Patients with preserved LVEF were included if they had CHF symptoms, a prior hospitalization for CHF or objective CHF signs confirmed by chest X-ray and/or echocardiography. Patients were excluded if they had recent acute coronary syndrome or severe valvular disease amenable for surgical repair. Patients with atrial fibrillation, flutter, paced rhythm and absence of ventricular ectopic beats were excluded due to restrictions for reliable calculation of ECG markers [16, 17]. Then, the final sample where all ECG indices could be calculated was composed of 597 patients. All patients signed informed consent and the study protocol was approved by all the institutional investigation committees from the following participant hospitals: Valme Hospital (Dr Juan Leal del Ojo, Dr Antonio Fernández, and Dr Dolores García-Medina), Santiago de Compostela Hospital (Dr Pilar Mazón), Son Dureta Hospital (Dr Miquel Fiol and Dr Carlos Fernández), Arrixaca Hospital (Dr Mariano Valdés), Gregorio Marañon Hospital (Dr Roberto Muñoz, Dr Jesús Almendral and Dr Marta Dominguez), Joan XXIII Hospital (Dr Alfredo Bardají and Dr Pilar Valdovinos), Insular Las Palmas Hospital (Dr Vicente Nieto, Dr Ricardo Huerta), Sant pau Hospital (Dr Agustina Castellví-Grisó, Dr Maite Domingo, and Dr Mariana Noguero) [13].

Follow-up visits were conducted on an outpatient basis every 6 months, for a median of 44 months. Primary endpoints were SCD and PFD. Cardiac death (CD) was divided into SCD and PFD. Death was defined as SCD if it was: (i) a witnessed death occurring within 60 min from the onset of new symptoms, unless a cause other than cardiac was obvious; (ii) an unwitnessed death (<24h) in the absence of preexisting progressive circulatory failure or other causes of death; or (iii) a death during attempted resuscitation. Deaths occurring in hospitals as a result of refractory progressive end-stage CHF, or CHF patients undergoing heart transplantation, were defined as PFD. Endpoints were reviewed and classified by the MUSIC Study Endpoint Committee [13].

### Clinical and ECG markers

The clinical variables used in this study are listed in Table 1. ECG markers reflecting spatio-temporal dispersion of repolarization (IAA), repolarization restitution (Δα^QT^, Δα^Tpe^, TMR) and sympathovagal balance (TS) were computed and used to develop the risk models, together with other commonly used ECG variables (see Table 1). Detailed descriptions of IAA, Δα^QT^, Δα^Tpe^, TMR and TS are presented in Table 2. Previous studies on this dataset have shown that IAA is associated with SCD when dichotomized at 3.7μV [16] and TS predicts SCD and PFD when dichotomized at 2.5 ms/RR [17]. Δα^QT^ and Δα^Tpe^ have been shown to be associated with SCD when dichotomized at 0.228 and 0.028, respectively [18, 19], while Δα^Tpe^ has been shown to predict PFD when dichotomized at 0.022 [19]. TMR, a novel index of T-wave morphology restitution, was calculated by time-warping the morphology of the T-waves [20] at different RR interval values and was found to predict SCD when dichotomized at TMR = 0.040 [21].

**Table 1:** Characteristics of patients according to their outcome. Data are represented as median (interquartile range) for continuous variables and as number (percentage) for dichotomized variables.

Variable	Survivors and non-CD victims (n = 486)	SCD (n = 49)	PFD (n = 62)
**Clinical variables**			
Age [years]	63 (18) †	67 (13)	69 (15) †
Male gender	323 (70%)	41 (84%) †	46 (74%)
Diabetes	163 (35%) †	20 (41%)	33 (53%) †
NYHA class III	62 (13%) †	14 (29%) †	21 (34%) †
Ischemic etiology	220 (48%) †	28 (57%)	37 (60%)
ARB or ACE inhibitors	419 (91%)	40 (82%)	51 (82%)
Beta-blockers	337 (73%) †	36 (74%)	35 (57%) †
Amiodarone	32 (7%) †	6 (12%)	8 (13%)
LVEF≤35%	238 (51%) †	36 (74%) †	42 (68%) †
LVEF [%]	35 (16) †	30 (16) †	30 (15) †
**ECG variables**			
Median RR [s]	0.86 (0.18)	0.85 (0.21)	0.84 (0.21)
RR range [s]	0.43 (0.19) †	0.37 (0.27)	0.35 (0.16)†
QRS>120 ms	184 (40%)	23 (47%)	28 (45%)
CIA	105 (23%) †	18 (37%)	24 (39%) †
Δα^Tpe^≥0.036	142 (31%)	27 (55%) †	14 (23%)
Δα^Tpe^≤0.022	206 (45%)	14 (29%) †	39 (63%) †
Δα^Tpe^ [adim.]	0.024 (0.03)	0.039 (0.04) †	0.019 (0.03)
Δα^QT^≥0.228	152 (33%)	24 (49%) †	21 (34%)
Δα^QT^ [adim.]	0.197 (0.09) †	0.216 (0.10)	0.205 (0.11)
IAA≥3.7μV	100 (22%) †	20 (41%) †	15 (24%)
IAA [μV]	2.921 (1.18)	3.207 (2.21)	2.758 (1.31)
TS≤2.5 ms/RR	186 (40%) †	33 (67%) †	49 (79%) †
TS [ms/RR]	3.250 (4.53)†	1.597 (4.28) †	1.245 (1.61) †
TMR≥0.040	208 (45%) †	35 (71%) †	26 (42%)
TMR [adim.]	0.038 (0.02)†	0.046 (0.03) †	0.037 (0.03)

ACE: Angiotensin-Converting Enzyme; ARB: Angiotensin Receptor Blocker; CIA = complex index of arrhythmia; IAA = Index of Average Alternans; LVEF = Left Ventricular Ejection Fraction; NYHA = New York Heart Association; PFD = Pump Failure Death; SCD = Sudden Cardiac Death; TMR = T-wave Morphology Restitution; TS = Turbulence Slope;

^†^ p<0.05 for comparison against the group formed by the other outcomes.

**Table 2:** ECG variables used for SCD or PFD risk prediction.

ECG marker	Short methodological description	Mechanism	References
IAA	First, selection of signal segments suitable for automatic analysis (128 beats with a 50% overlap between adjacent segments). Then, estimation of T-wave alternans amplitude in those segments with a multi-lead scheme that combines periodic component analysis with the Laplacian likelihood ratio method. Finally, computation of the average of all segment’s T-wave alternans amplitudes.	Average T-wave alternans activity in 24-h	[16]
TS	Maximum positive slope of a regression line assessed over any of 5 consecutive RR intervals within the first 20 sinus RR intervals after a VPB.	Initial phase of sinus rhythm deceleration	[17]
Δ*α*^*QT*^	Derivative of the QT interval with respect to a surrogate of the RR interval that accounts for the QT memory dependence on RR.	Ventricular depolarization and repolarization restitution	[18]
Δ*α*^*Tpe*^	Derivative of the Tpe interval with respect to a surrogate of the RR interval that accounts for the Tpe memory dependence on RR.	Dispersion of repolarization restitution	[19]
TMR	First, calculation of the histogram of the RR series and division of the histogram into 10 ms wide pairs of bins distributed symmetrically around the median, and exclusion of those bins with less than 50 values. Next, calculation of a mean warped T-wave for the two bins in the pair with the highest separation in RR from the remaining ones. Then, quantification of the morphological variability between both signal-averaged T-waves [20]. Finally, TMR was defined as the morphological variability, normalized by the difference between the longest and shortest RR.	T-wave morphological change per RR range increment.	[21]

IAA = Index of Average Alternans; TMR = T-wave Morphology Restitution; TS = Turbulence Slope; VPB = Ventricular Premature Beat.

### Statistical analyses

The primary outcomes of interest were time to SCD and time to PFD, calculated from the time of enrollment in the MUSIC study. Patients who died from causes not included in the endpoints or from competing risks were censored at the time of death.

As a first step in the development of the risk models, univariable analyses using Cox regression were performed in the sample population in order to determine the relationship between each potential risk marker and SCD or PFD. Multivariable Cox regression analyses were subsequently performed, with risk markers significantly associated with outcomes in the univariable analysis being selected and placed into backward stepwise elimination models and risk markers associated with p>0.05 being eliminated from the models. Three different models were fit for SCD and PFD separately: a clinical model (including clinical variables only), an ECG-based model (including ECG markers only) and a combined model (combining clinical and ECG markers). Finally, a point scoring system was constructed in which points were assigned to each marker using beta-coefficients from each of the final multivariable Cox regression models [22, 23, 24]. A risk score was calculated for each patient by adding the points associated with each marker (see S1 Appendix). For each risk model, three risk groups were identified based on the distribution of the scores: low-risk (first and second quintiles), middle-risk (third and fourth quintiles) and high-risk (fifth quintile). Association between SCD risk groups and the SCD outcome was evaluated using the survival probability estimated by the Kaplan-Meier method using the log-rank test. Association between PFD risk groups and the PFD outcome was evaluated in the same way. Hazard ratios (HRs) were calculated using the low-risk group as a reference. A p-value <0.05 was considered as statistically significant. Statistical analysis was performed using SPSS version 22.0 (SPSS, Inc. Chicago IL).

The area under the receiver operating curve (ROC), denoted by AUC, was calculated by considering the Sensitivity and Specificity of the three risk models (clinical, ECG and combined) in identifying patients associated with a specific outcome (SCD or PFD) at any time during the follow-up.

## Results

### Clinical characteristics and cardiac events

There were 425 men and 172 women in the sample population (aged 18–89, 63 ± 12 years). The majority of patients (83%) were in NYHA class II, while the remaining 17% were in NYHA class III and LVEF was 37% ± 14%. The detailed characteristics of the study population are shown in Table 1. During follow-up, there were 134 deaths (22%), including 111 CD (19%) and 23 non-CD (4%). Among the 111 CDs, 49 were SCD and 62 were PFD.

### Associations with SCD and PFD

SCD victims were more frequently men (p = 0.048), were in NYHA class III (p = 0.047) and had LVEF≤35% (p = 0.010), while PFD victims were more likely older than the rest of patients (p = 0.013), were more frequently diabetic (p = 0.009), in NYHA class III (p = 0.001), were not treated with beta-blockers (p = 0.012) and also had low LVEF (p = 0.044). Ischemic etiology was not associated with SCD or PFD (Table 1).

ECG analysis showed that SCD victims were associated with higher Δα^Tpe^ (p = 0.002), Δα^QT^ (p = 0.041), IAA (p = 0.008) and TMR (p = 0.001) and lower TS (p = 0.004). PFD victims were associated with lower RR range (p<0.001), Δα^Tpe^ (p = 0.003), TS (p<0.001) and a higher rate of a complex index of arrhythmia (CIA) composed of non-sustained ventricular tachycardia (NSVT) and more than 240 ventricular premature beats (VPBs) in 24 h (p = 0.014). QRS duration was not associated with SCD or PFD.

### Predictors of SCD and PFD

The definition of the dichotomized variables introduced in the Cox analysis is presented in S1 Table. In the univariable analysis, SCD was associated with male gender, NYHA class III, LVEF≤35%, Δα^Tpe^≥0.028, Δα^QT^≥0.228, IAA≥3.7μV, TS≤2.5 ms/RR and TMR≥0.04 (Table 3), while PFD was associated with age, diabetes, NYHA class III, absence of treatment with beta-blockers, LVEF≤35%, reduced RR range, low rate of CIA, Δα^Tpe^≤0.022 and TS≤2.5 ms/RR (Table 4).

**Table 3:** Univariable predictors of SCD.

Risk markers	Univariable	
	HR (95% CI)	*p*
Male gender (*x*_*g*_ = 1)	2.159 (1.012–4.606)	0.046
NYHA class III (*x*_*NYHA*_ = 1)	2.189 (1.177–4.071)	0.013
LVEF≤35% (*x*_*LVEF*_ = 1)	2.335 (1.238–4.403)	0.009
LVEF [per 1 SD increment]	0.576 (0.402–0.824)	0.003
Δα^Tpe^≥0.028 ($x_{\Delta \alpha_{Tpe}^{SCD}}=1$)	2.676 (1.524–4.700)	0.001
Δα^QT^≥0.228 ($x_{\Delta \alpha_{QT}}=1$)	1.921 (1.097–3.364)	0.022
IAA≥3.7μV (*x*_*IAA*_ = 1)	2.335 (1.321–4.128)	0.004
TS≤2.5ms/RR (*x*_*TS*_ = 1)	2.641 (1.453–4.802)	0.001
TMR≥0.04 (*x*_*TMR*_ = 1)	2.929 (1.576–5.445)	0.001
Δα^Tpe^ [per 1SD increment]	1.501 (1.223–1.844)	<0.001
TS [per 1 SD increment]	0.505 (0.297–0.857)	0.011
TMR [per 1 SD increment]	1.466 (1.235–1.741)	<0.001

HR = Hazard ratio; NYHA = New York Heart Association; LVEF = Left Ventricular Ejection Fraction; IAA = Index of Average Alternans; TS = Turbulence Slope; TMR = T-wave Morphology Restitution.

**Table 4:** Univariable predictors of PFD.

Risk marker	Univariable	
	HR (95% CI)	*p*
Age [per 1 SD increment]	1.378 (1.047–1.813)	0.022
Diabetes (*x*_*Diab*_ = 1)	2.011 (1.221–3.312)	0.006
NYHA class III (*x*_*NYHA*_ = 1)	2.892 (1.709–4.896)	<0.001
Beta-blockers (*x*_*β*_ = 1)	0.498 (0.302–0.823)	0.007
LVEF≤35% (*x*_*LVEF*_ = 1)	1.792 (1.052–3.053)	0.032
RR range [per 1 SD increment]	0.587 (0.451–0.764)	<0.001
CIA (*x*_*CIA*_ = 1)	2.034 (1.220–3.391)	0.006
Δα^Tpe^≤0.022 ($x_{\Delta \alpha_{Tpe}^{PFD}}=1$)	2.068 (1.235–3.462)	0.006
TS≤2.5ms/RR (*x*_*TS*_ = 1)	4.975 (2.698–9.172)	<0.001
TS [per 1 SD increment]	0.410 (0.242–0.696)	0.001

CIA = complex index of arrhythmia; HR = Hazard ratio; SD = Standard Deviation; NYHA = New York Heart Association; LVEF = Left Ventricular Ejection Fraction; TS = Turbulence Slope.

### Multivariable models

For SCD prediction, all the clinical variables with significant association in the univariable analysis remained significant in the multivariable clinical model. Δα^QT^≥0.228 was no longer significant in the multivariable ECG model. The multivariable model combining clinical and ECG markers included male gender, NYHA class III, LVEF≤35%, Δα^Tpe^>0.036, IAA>3.7μV and TMR>0.04. Then, TS≤2.5 ms/RR was eliminated after adjusting for clinical variables (Table 5). The equations of the three SCD risk models are described in S2 Appendix.

**Table 5:** Multivariable predictors of SCD.

Risk markers	Clinical Multivariable			ECG Multivariable			Combined Multivariable		
	HR (95% CI)	β	*p*	HR (95% CI)	β	*p*	HR (95% CI)	β	*p*
Male gender (*x*_*g*_ = 1)	2.248 (1.050–4.814)	0.810	0.037	-	-	-	2.750 (1.276–5.927)	1.012	0.010
NYHA class III (*x*_*NYHA*_ = 1)	2.221 (1.189–4.150)	0.798	0.012	-	-	-	2.499 (1.328–4.702)	0.916	0.005
LVEF≤35% (*x*_*LVEF*_ = 1)	2.165 (1.146–4.092)	0.772	0.017	-	-	-	1.997 (1.052–3.792)	0.692	0.035
Δα^Tpe^≥0.028 ($x_{\Delta \alpha_{Tpe}^{SCD}}=1$)	-	-	-	2.365 (1.329–4.210)	0.861	0.003	2.550 (1.440–4.515)	0.936	0.001
Δα^QT^≥0.228 ($x_{\Delta \alpha_{QT}}=1$)	-	-	-	N.S.	N.S.	N.S.	N.S.	N.S.	N.S.
IAA≥3.7μV (*x*_*IAA*_ = 1)	-	-	-	2.377 (1.339–4.221)	0.866	0.003	2.271 (1.278–4.035)	0.820	0.005
TS≤2.5ms/RR (*x*_*TS*_ = 1)	-	-	-	2.180 (1.193–3.986)	0.780	0.011	N.S.	N.S.	N.S.
TMR≥0.04 (*x*_*TMR*_ = 1)	-	-	-	2.193 (1.162–4.140)	0.785	0.015	2.883 (1.531–5.429)	1.059	0.001

HR = Hazard ratio; NYHA = New York Heart Association; LVEF = Left Ventricular Ejection Fraction; NSVT = Non-Sustained Ventricular Tachycardia; VPB = Ventricular Premature Beat; IAA = Index of Average Alternans; TS = Turbulence Slope; TMR = T-wave Morphology Restitution; N.S. = Not Significant, N.A. = Not Applicable.

For PFD risk prediction, the multivariable clinical model included all clinical variables except for age (Table 6). The variable CIA (NSVT and >240 VPBs in 24 h) was eliminated from the multivariable ECG model. The multivariable model combining clinical and ECG markers included diabetes, NYHA class III, Δα^Tpe^≤0.022 and TS≤2.5ms/RR. Then, absence of treatment with beta-blockers, LVEF≤35% and reduced RR range were eliminated when adjusting for both clinical and ECG variables (Table 6). The equations of the three PFD risk models are described in S2 Appendix.

**Table 6:** Multivariable predictors of PFD.

Risk markers	Clinical multivariable			ECG multivariable			Combined multivariable		
	HR (95% CI)	β	*p*	HR (95% CI)	β	*p*	HR (95% CI)	β	*p*
Age [per 1 SD increment]	N.S.	N.S.	N.S.	-	-	-	N.S.	N.S.	N.S.
Diabetes (*x*_*Diab*_ = 1)	1.842 (1.112–3.049)	0.611	0.018	-	-	-	1.697 (1.022–2.818)	0.529	0.041
NYHA class III (*x*_*NYHA*_ = 1)	2.305 (1.342–3.959)	0.835	0.002	-	-	-	1.972 (1.154–3.370)	0.679	0.013
Beta-blockers (*x*_*β*_ = 1)	1.859 (1.118–3.091)	0.620	0.017	-	-	-	N.S.	N.S.	N.S.
LVEF≤35% (*x*_*LVEF*_ = 1)	1.768 (1.034–3.026)	0.570	0.037	-	-	-	N.S.	N.S.	N.S.
RR range [per 1 SD increment]	-	-	-	0.753 (0.566–1.000)	-0.284	0.050	N.S.	N.S.	N.S.
CIA (*x*_*CIA*_ = 1)	-	-	-	N.S.	N.S.	N.S.	N.S.	N.S.	N.S.
Δα^Tpe^≤0.022 ($x_{\Delta \alpha_{Tpe}^{PFD}}=1$)	-	-	-	2.174 (1.298–3.642)	0.777	0.003	2.219 (1.320–3.730)	0.797	0.003
TS≤2.5ms/RR (*x*_*TS*_ = 1)	-	-	-	4.132 (2.165–7.884)	1.419	<0.001	4.160 (2.225–7.779)	1.425	<0.001

CIA = complex index of arrhythmia; HR = Hazard ratio; SD = Standard Deviation; NYHA = New York Heart Association; ARB: Angiotensin Receptor Blocker; ACE: Angiotensin-Converting Enzyme; LVEF = Left Ventricular Ejection Fraction; TS = Turbulence Slope; N.S. = Not Significant; N.A. = Not Applicable.

### SCD and PFD prediction

**Fig 1:**
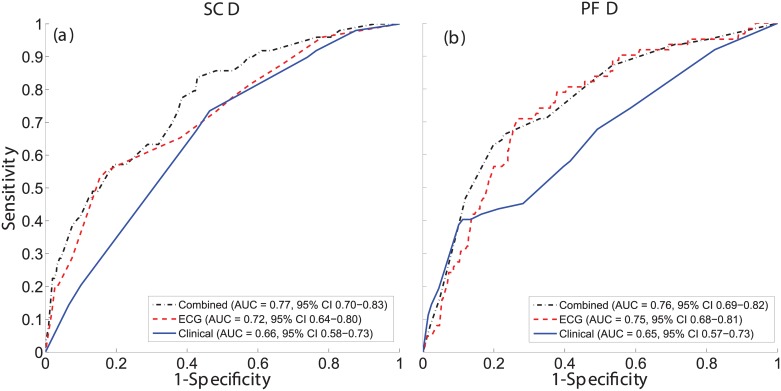
ROC curves of the clinical, ECG and combined specific risk models for SCD and PFD classification. ROC curves and AUCs for the clinical (solid blue), ECG (dashed red), and combined (dotted black) prediction models in the classification of SCD (a) and PFD (b) victims.

According to ROC analysis, ECG markers provided a more accurate prediction of both SCD and PFD with respect to clinical markers (Fig 1). Accuracy further increased for SCD prediction when combining clinical and ECG markers.

**Fig 2:**
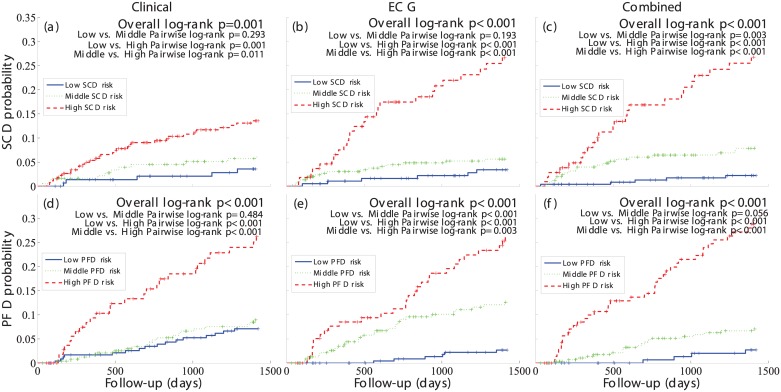
SCD and PFD probability curves of the clinical, ECG and combined specific risk models. Probability curves of the three risk groups, low (solid blue), middle (dotted green) and high (dashed red) defined in the clinical (left), ECG (middle) and combined (right) specific risk models for SCD (top) and PFD (bottom).

Kaplan-Meier analysis showed that SCD probability for the high-risk group was higher in the ECG model than in the clinical model and it further increased in the combined model (Fig 2(a)–2(c)). Moreover, in the combined model, SCD probability for the low-risk group was lower than in the ECG and clinical models, therefore further increasing the distance between low and high-risk curves. Regarding PFD, the distance between low- and high-risk groups was significant for all three models, but larger for the combined one (Fig 2(d)–2(f)).

**Fig 3:**
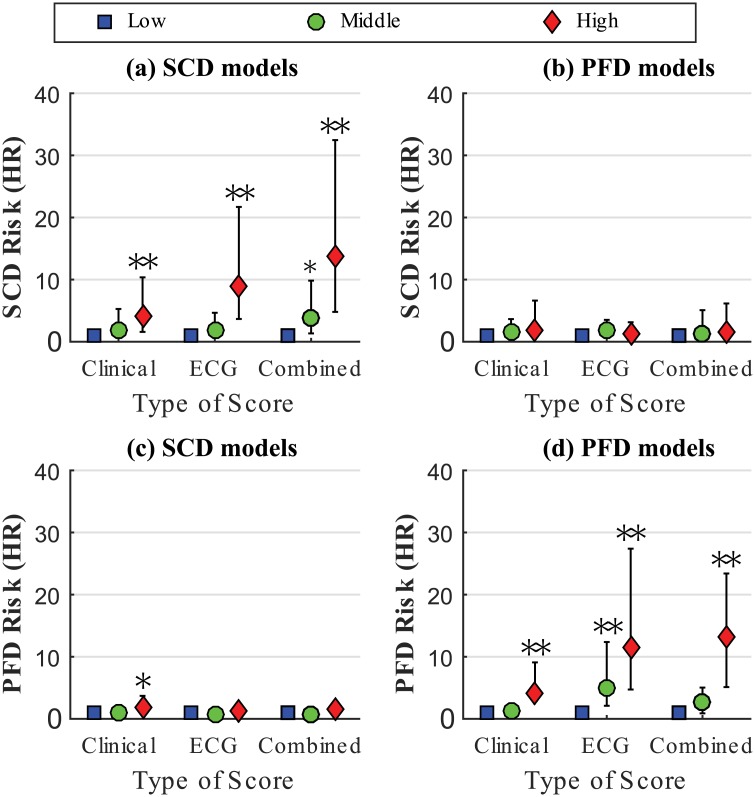
SCD and PFD hazard ratios of the clinical, ECG and combined specific risk models. Hazard ratios of SCD ((a) and (b)) and PFD ((c) and (d)) for the three risk groups, low (blue square), middle (green circle) and high (red diamond) defined in the clinical, ECG and combined specific models. * and **indicate p<0.05 and p<0.005 with respect to the low risk group, respectively.

Both SCD and PFD prediction improved when clinical and ECG markers where integrated into the combined model. HRs for SCD was equal to 4.0, 8.9 and 13.8 for clinical, ECG and combined models, respectively (Fig 3(a)), and HRs for PFD equal to 4.1, 11.4 and 13.1 for clinical, ECG and combined models, respectively (Fig 3(d)). Importantly, models designed to predict SCD did not predict PFD and models designed to predict PFD did not predict SCD (Fig 3(b) and 3(c)), therefore demonstrating specificity, on top of sensitivity, in the prediction of the designated mode of death.

### Analyses in reduced and preserved LVEF populations

The number of SCD victims was significantly higher in the HFrEF group than in the HFpEF group (11% vs 5%, p = 0.010). In the HFrEF group, the number of PFD victims was also significantly higher than in the HFpEF group (13% vs 8%, p = 0.044).

In the HFrEF group, SCD victims were less frequently under ARB or ACE inhibitors (p = 0.027) and were associated with higher Δα^Tpe^ (p = 0.008) and TMR (p = 0.001) but lower TS (p = 0.025), while PFD victims were more frequently diabetic (p = 0.001), in NYHA class III (p = 0.001) and showed lower RR range (p = 0.003), Δα^Tpe^ (p = 0.005) and TS values (p<0.001) but a higher rate of CIA (p = 0.030). Univariable Cox analyses showed that the only clinical variable significantly associated with SCD in the HFrEF group was administration of ARB or ACE inhibitors (S2 Table). Hence, the clinical model included just this single variable. Univariable results for clinical and ECG variables for PFD prediction are shown in S3 Table. The ECG variables that remained significantly associated with SCD in a multivariable Cox analysis and were included in the ECG model were Δα^Tpe^≥0.028, IAA≥3.7μV, TS≤2.5 ms/RR and TMR≥0.04 (S4 Table), as in the general population analysis. The AUC of the ECG model was 0.72 (0.63–0.81), p<0.001. The combined model for SCD included the four above-mentioned ECG-derived variables and administration of ARB or ACE inhibitors (S4 Table) (AUC of 0.73 (0.64–0.81), p<0.001). Based on multivariable Cox regression analysis for PFD in the HFrEF group, the clinical model included diabetes, NYHA class III and administration of ARB or ACE inhibitors (AUC of 0.69 (0.60–0.78), p<0.001), the ECG model included reduced RR range, Δα^Tpe^≤0.022 and TS≤2.5 ms/RR (S5 Table) (AUC of 0.76 (0.68–0.83), p<0.001), while the combined model included the three variables from the clinical model, Δα^Tpe^≤0.022 and TS≤2.5 ms/RR (S5 Table) (AUC of 0.79 (0.71–0.86), p<0.001).

When dividing the HFrEF group into three risk groups according to the models, SCD prediction improved when clinical and ECG markers where integrated into the combined model. However, the combined model for PFD showed lower predictive power than the ECG model. Additionally, the combined model designed to predict SCD also predicted PFD and vice versa.

In the HFpEF group, SCD victims were associated with a higher rate of CIA (p = 0.024) and higher values of Δα^QT^ (p = 0.028), while PFD victims were characterized by higher age (p = 0.012) and lower values of TS (p = 0.005). Univariable Cox analyses showed that no clinical variable was significantly associated with SCD or PFD (S2 and S3 Tables). Consequently, no clinical models for either SCD or PFD were obtained in this population (S4 Table). The multivariable ECG model for SCD consisted of the variable CIA and Δα^QT^≥0.228 (AUC of 0.72 (0.55–0.89), p = 0.008). The ECG model for PFD included only TS≤2.5 ms/RR, as this was the only variable significantly associated with PFD along follow-up. For SCD, no multivariable combined model could be calculated. The multivariable combined model for PFD included age and TS (S4 and S5 Tables) (AUC of 0.64 (0.51–0.78), p = 0.033).

When dividing the HFpEF group into three risk groups, the high SCD risk group in the ECG-derived model was significantly associated with SCD (HAR 2.219 (1.217–4.045), p = 0.009), with no significant association with PFD. Also, the high PFD risk group in the combined model was significantly associated with PFD (HAR 2.578 (1.453–4.573), p = 0.001), with no significant association with SCD.

## Discussion

The main result of this study is that in mild-to-moderate CHF patients the combination of clinical and ECG markers significantly improves prediction of both SCD and PFD, as compared to the use of clinical variables only. This indicates a possible new strategy to identify CHF patients specifically at risk of SCD or PFD.

In a combined model, the clinical variables that predicted SCD were male gender, NYHA class III and LVEF≤35%. Previous studies have also shown that men have higher SCD risk than women [25, 26], while the contribution of NYHA class to SCD risk is still unclear [27, 28]. Impaired LVEF is at present the only risk factor considered for ICD implantation in high SCD risk patients, but its specificity is insufficient [29].

Diabetes, NYHA class III, absence of treatment with beta-blockers and LVEF≤35% predicted PFD in a multivariable clinical model. These results confirm previous findings reporting the relation between end-stage CHF and low cardiac output and LVEF, diabetes due to increased congestion as well as advanced stages of NYHA class [30]. Also, treatment with beta-blockers or limiting neuro-hormonal activation has been shown to be especially important in delaying CHF progression [31].

The ECG variables that independently predicted SCD in the ECG model were Δα^Tpe^, IAA, TS and TMR. Our results confirm that SCD risk is associated with increased dispersion of repolarization restitution [32, 33], increased variability of temporal dispersion of repolarization [34] and baroreceptor-heart rate reflex sensitivity [17, 35]. More importantly, our results confirm the hypothesis that a combination of ECG markers capturing complementary information about arrhythmic substrates could improve SCD prediction. Future studies could include additional risk indices into the proposed models.

The ECG markers that independently predicted PFD in the ECG model were Δα^Tpe^, TS and the range of RR. This indicates that PFD is also characterized by baroreceptor-heart rate reflex sensitivity [17, 35]. However, lower values of Δα^Tpe^ indicative of higher PFD risk suggest that PFD victims experience a reduction in the ability of the ventricles to adapt to changes in heart rate, as opposed to SCD victims [36].

The ECG models for SCD and PFD showed better prognostic value than the clinical models. The combination of clinical and ECG markers synergistically improved the prognostic value for both SCD and PFD. For PFD prediction the improvement achieved by combining clinical and ECG markers was only marginal with respect to the results of the ECG model. This suggests that clinical variables do not add complementary information to ECG markers for PFD risk prediction. More importantly, the ECG and combined risk models demonstrated high sensitivity (association with the designated mode of death) and specificity (no association with the alternative mode of death) for SCD and PFD prediction, while the clinical risk model for SCD prediction lacked specificity and predicted PFD in addition to SCD.

Separately considering HFrEF and HFpEF subpopulations, the number of SCD and PFD victims was significantly higher in the HFrEF group than in the HFpEF group, supporting previous studies [37]. A larger number of ECG-derived markers were predictive of SCD or PFD in HFrEF patients as compared to HFpEF patients. Still, in this work we found that an ECG-derived model including a higher rate of non-sustained ventricular arrhythmia and enhanced spatio-temporal inhomogeneity of ventricular repolarization (higher Δ*α*^*QT*^) demonstrated specific SCD predictive value in the HFpEF group. These results support previous studies where ECG-derived arrhythmic markers were found to be predictive of SCD in patients with preserved LVEF [38]. For PFD prediction, a multivariable combined model including age and TS predicted PFD in this group, supporting previous results in the literature where autonomic markers have shown capacity to predict PFD in patients with preserved LVEF [39, 40]. Overall, our results in the HFrEF group were very similar to those in the general study population, with the exception of the individual association of the clinical variables with SCD, since only administration of ARB or ACE inhibitors showed predictive value in the HFrEF group. In a combined model including clinical variables and ECG-derived markers for PFD risk prediction, the autonomic index TS was included as well when analyzing the HFrEF subpopulation, in agreement with previous studies in the literature investigating mortality due to pump failure in patients with reduced LVEF [41].

### Limitations

Due to the low number of SCD and PFD victims, a division of the sample population into training and test groups for validation of the results was not performed. Moreover, the proposed risk scores were calculated in patients in sinus rhythm, which limits its applicability, especially in patients with reduced LVEF, where the incidence of AF is significant. Further studies may consider modifications of ECG markers to assess the proposed models in CHF patients with other rhythms. The assessment of clinical and combined models integrating additional variables deserves further investigation. The results obtained in this work are not directly comparable with those of a previous publication reporting clinical scores on the MUSIC study [13], because the sample population analyzed in the present paper is limited to CHF patients in sinus rhythm. Also, since only a Holter ECG recording per patient was available, reproducibility was not studied in the present work. In addition, we found more SCD and PFD victims with NYHA class III, as compared to NYHA class II, supporting previously published results [42]. However, it should be noted that the evaluation of the relationship between NYHA class and SCD or PFD was limited in this study since only CHF patients in NYHA classes II and III were available in the population. Finally, the prognostic discriminative power of the clinical models may be reduced because the clinical indices available for this study did not include information about neuro-hormonal activation, natriuretic peptides, diastolic function or peak oxygen consumption. The inclusion of these other indices may to some extent impact the prediction of SCD and, most likely, PFD.

## Conclusion

This study demonstrates that two risk prediction models combining clinical and ECG markers of electrophysiological and autonomic abnormalities specifically predict SCD and PFD. For SCD, the combination of clinical and ECG markers substantially improved risk prediction as compared to the use of only clinical or ECG markers. For PFD, the use of only ECG markers demonstrated its superiority over the use of clinical markers. The combination of clinical and ECG variables only marginally improved the PFD predictive value of the ECG model.

## Supporting information

S1 Appendix Construction of the models. (DOCX)

S2 Appendix Final models. (DOCX)

S1 Table Definition of the dichotomized variables used to build SCD and PFD risk models. (DOCX)

S2 Table Univariable predictors of SCD in both reduced and preserved LVEF populations. (DOCX)

S3 Table Univariable predictors of PFD in both reduced and preserved LVEF populations. (DOCX)

S4 Table Multivariable predictors of SCD in both reduced and preserved LVEF populations. (DOCX)

S5 Table Multivariable predictors of PFD in both reduced and preserved LVEF populations. (DOCX)

S6 Table Clinical and ECG data. (XLSX)
