# Supplementary material for: Sudden cardiac death and pump failure death prediction in chronic heart failure by combining ECG and clinical markers in an integrated risk model
Source: PLoS One. 2017 Oct 11;12(10):e0186152. doi: 10.1371/journal.pone.0186152 (PMC5636125; doi:10.1371/journal.pone.0186152)
Supplement: S1 Appendix — (DOCX) [file pone.0186152.s001.docx]

**Supporting information**

**S1 Appendix: Construction of the models**

SCD and PFD prediction models were defined according to the following equation:

$\mathcal{S}_{M}^{E}=\sum_{i=1}^{I} \beta_{i}x_{i}$,

where $\mathcal{S}$ denotes the score, $M$ denotes the model, either the clinical (“Cli”), the ECG-based (“ECG”), or the combined (“Com”); $E$ denotes the endpoint, SCD or PFD; $I$ is the number of dichotomized variables retained in the multivariable model; $\beta_{\boldsymbol{i}}$ is the coefficient of the i-th dichotomized variable; and$x_{i}$is the i-th dichotomized variable (i.e. $x_{i}$takes the value 0 when the variable is below the defined cutoff point and 1 when it is above, see S1 Table).
