# Supplementary material for: Sudden cardiac death and pump failure death prediction in chronic heart failure by combining ECG and clinical markers in an integrated risk model
Source: PLoS One. 2017 Oct 11;12(10):e0186152. doi: 10.1371/journal.pone.0186152 (PMC5636125; doi:10.1371/journal.pone.0186152)
Supplement: S2 Appendix — (DOCX) [file pone.0186152.s002.docx]

**S2 Appendix: Final models**

SCD prediction models were calculated as follows:

Clinical:

$$\mathcal{S}_{Cli}^{SCD}=0.810*x_{g}+0.798*x_{NYHA}+0.772*x_{LVEF}$$

ECG:

$$\mathcal{S}_{ECG}^{SCD}=0.861*x_{{\Delta\alpha}_{Tpe}^{SCD}}+0.866*x_{IAA}+0.780*x_{TS}+0.785*x_{TMR}$$

Combined:

$$\mathcal{S}_{Com}^{SCD}=1.012*x_{g}+0.916*x_{NYHA}+0.692*x_{LVEF}+0.936*x_{{\Delta\alpha}_{Tpe}^{SCD}}+0.810*x_{IAA}+1.059*x_{TMR}.$$

PFD prediction models were:

Clinical:

$$\mathcal{S}_{Cli}^{PFD}=0.611*x_{Diab}+0.835*x_{NYHA}+0.620*x_{\beta}+0.570*x_{LVEF}$$

ECG:

$$\mathcal{S}_{ECG}^{PFD}=-0.284*\Delta RR \left[ per 1 SD increment \right]+0.777*x_{{\Delta\alpha}_{Tpe}^{PFD}}+1.419*x_{TS}$$

Combined:

$$\mathcal{S}_{Com}^{PFD}=0.529*x_{Diab}+0.679*x_{NYHA}+0.797*x_{{\Delta\alpha}_{Tpe}^{PFD}}+1.425*x_{TS}.$$
