# Supplementary material for: Sudden cardiac death and pump failure death prediction in chronic heart failure by combining ECG and clinical markers in an integrated risk model
Source: PLoS One. 2017 Oct 11;12(10):e0186152. doi: 10.1371/journal.pone.0186152 (PMC5636125; doi:10.1371/journal.pone.0186152)
Supplement: S1 Table — (DOCX) [file pone.0186152.s003.docx]

**S1 Table: Dichotomized variables used to build SCD and PFD risk scores.**

| **Dichotomized variable** | **Definition** |
| --- | --- |
| $x_{g}$ | 1, if male gender; 0 otherwise |
| $x_{NYHA}$ | 1, if NYHA class III; 0 otherwise |
| $x_{Diab}$ | 1, if diabetic; 0 otherwise |
| $x_{\beta}$ | 1, if treated with beta-blockers; 0 otherwise |
| $x_{LVEF}$ | 1, if LVEF≤35%; 0 otherwise |
| $x_{CIA}$ | 1, if presence of NSVT and >240 VPBs/24-h; 0 otherwise |
| $x_{{\Delta\alpha}_{Tpe}^{SCD}}$ | 1, if Δα^Tpe^≥0.028; 0 otherwise |
| $x_{{\Delta\alpha}_{Tpe}^{PFD}}$ | 1, if Δα^Tpe^≤0.022; 0 otherwise |
| $x_{{\Delta\alpha}^{QT}}$ | 1, if ${\Delta\alpha}^{QT}$≥0.228; 0 otherwise |
| $x_{IAA}$ | 1, if IAA≥3.7µV; 0 otherwise |
| $x_{TS}$ | 1, if TS≤2.5ms/RR; 0 otherwise |
| $x_{TMR}$ | 1, if TMR≥0.040; 0 otherwise |

CIA = complex index of arrhythmia; IAA = Index of Average Alternans; LVEF = Left Ventricular Ejection Fraction; NSVT = Non-Sustained Ventricular Tachycardia; NYHA = New York Heart Association; PFD = Pump Failure Death; SCD = Sudden Cardiac Death; TMR = T-wave Morphology Restitution; TS = Turbulence Slope; VPB = Ventricular Premature Beat.
