# Supplementary material for: Sudden cardiac death and pump failure death prediction in chronic heart failure by combining ECG and clinical markers in an integrated risk model
Source: PLoS One. 2017 Oct 11;12(10):e0186152. doi: 10.1371/journal.pone.0186152 (PMC5636125; doi:10.1371/journal.pone.0186152)
Supplement: S2 Table — (DOCX) [file pone.0186152.s004.docx]

**S2 Table: Univariable predictors of SCD in both reduced and preserved LVEF populations.**

| LVEF≤35% | **Univariable** | |
| --- | --- | --- |
|  | HR (95% CI) | *p* |
| ARB or ACE inhibitors ($x_{inh}$=1) | 0.305 (0.127-0.734) | 0.008 |
| Δα^Tpe^≥0.028 ($x_{{\Delta\alpha}_{Tpe}^{SCD}}$=1) | 2.662 (1.384-5.123) | 0.003 |
| Δα^Tpe^ [per 1 SD increment] | 1.418 (1.135-1.770) | 0.002 |
| IAA≥3.7µV ($x_{IAA}$=1) | 2.681 (1.393-5.158) | 0.003 |
| TS≤2.5ms/RR ($x_{TS}$=1) | 2.275 (1.096-4.720) | 0.027 |
| TMR≥0.04 ($x_{TMR}$=1) | 3.238 (1.561-6.714) | 0.002 |
| TMR [per 1 SD increment] | 1.659 (1.297-2.123) | <0.001 |

| LVEF>35% | **Univariable** | |
| --- | --- | --- |
|  | HR (95% CI) | *p* |
| CIA | 4.018 (1.349-11.967) | 0.012 |
| Δα^Tpe^≥0.028 ($x_{{\Delta\alpha}_{Tpe}^{SCD}}$=1) | 3.132 (1.024-9.575) | 0.045 |
| Δα^QT^≥0.228 ($x_{{\Delta\alpha}_{QT}^{SCD}}$=1) | 4.725 (1.545-14.450) | 0.006 |
| Δα^QT^ [per 1 SD increment] | 2.622 (1.364-5.039) | 0.004 |
| TMR [per 1 SD increment] | 1.386 (1.015-1.893) | 0.040 |

HR = Hazard ratio; NYHA = New York Heart Association; LVEF = Left Ventricular Ejection Fraction; NSVT = Non-Sustained Ventricular Tachycardia; VPB = Ventricular Premature Beat; IAA = Index of Average Alternans; TS = Turbulence Slope; TMR = T-wave Morphology Restitution
