# Supplementary material for: Sudden cardiac death and pump failure death prediction in chronic heart failure by combining ECG and clinical markers in an integrated risk model
Source: PLoS One. 2017 Oct 11;12(10):e0186152. doi: 10.1371/journal.pone.0186152 (PMC5636125; doi:10.1371/journal.pone.0186152)
Supplement: S3 Table — (DOCX) [file pone.0186152.s005.docx]

**S3 Table: Univariable predictors of PFD in both reduced and preserved LVEF populations.**

| LVEF≤35% | **Univariable** | |
| --- | --- | --- |
|  | HR (95% CI) | *p* |
| Diabetes ($x_{Diab}$=1) | 3.088 (1.656-5.759) | <0.001 |
| NYHA class III ($x_{NYHA}$=1) | 3.314 (1.788-6.142) | <0.001 |
| ARB or ACE inhibitors ($x_{Inh}$=1) | 0.341 (0.143-0.810) | 0.015 |
| Beta-blockers ($x_{\beta}$=1) | 0.487 (0.263-0.902) | 0.022 |
| Maximum HR [per 1 SD increment] | 0.696 (0.522-0.928) | 0.013 |
| RR range [per 1 SD increment] | 0.591 (0.430-0.813) | 0.001 |
| CIA  ($x_{CIA}$=1) | 1.987 (1.082-3.649) | 0.027 |
| Δα^Tpe^≤0.022 ($x_{{\Delta\alpha}_{Tpe}^{PFD}}$=1) | 2.572 (1.354-4.885) | 0.004 |
| TS≤2.5ms/RR ($x_{TS}$=1) | 5.389 (2.270-12.793) | <0.001 |
| TS [per 1 SD increment] | 0.182 (0.068-0.491) | 0.001 |

| LVEF>35% | **Univariable** | |
| --- | --- | --- |
|  | HR (95% CI) | *p* |
| Age [per 1 SD increment] | 2.093 (1.221-3.591) | 0.007 |
| TS≤2.5ms/RR ($x_{TS}$=1) | 3.924 (1.564-9.844) | 0.004 |

CIA = complex index of arrhythmia; HR = Hazard ratio; SD = Standard Deviation; NYHA = New York Heart Association; ARB: Angiotensin Receptor Blocker; ACE: Angiotensin-Converting Enzyme; LVEF = Left Ventricular Ejection Fraction; TS = Turbulence Slope
