# Supplementary material for: Sudden cardiac death and pump failure death prediction in chronic heart failure by combining ECG and clinical markers in an integrated risk model
Source: PLoS One. 2017 Oct 11;12(10):e0186152. doi: 10.1371/journal.pone.0186152 (PMC5636125; doi:10.1371/journal.pone.0186152)
Supplement: S4 Table — (DOCX) [file pone.0186152.s006.docx]

**S4 Table: Multivariable predictors of SCD in both reduced and preserved LVEF populations.**

| LVEF≤35% | **Clinical Multivariable** | | | **ECG Multivariable** | | | **Combined Multivariable** | | |
| --- | --- | --- | --- | --- | --- | --- | --- | --- | --- |
|  | HR (95% CI) | β | *p* | HR (95% CI) | β | *p* | HR (95% CI) | β | *p* |
| ARB or ACE inhibitors ($x_{inh}$=1) | 0.305 (0.127-0.734) | -1.187 | 0.008 | - | - | - | 0.402 (0.163-0.995) | -0.910 | 0.049 |
| Δα^Tpe^≥0.028 ($x_{{\Delta\alpha}_{Tpe}^{SCD}}$=1) | - | - | - | 2.211 (1.138-4.295) | 0.794 | 0.019 | 2.087 (1.272-5.588) | 0.736 | 0.032 |
| IAA≥3.7µV ($x_{IAA}$=1) | - | - | - | 2.540 (1.319-4.891) | 0.932 | 0.005 | 2.298 (1.177-4.489) | 0.832 | 0.015 |
| TS≤2.5ms/RR ($x_{TS}$=1) | - | - | - | N.S. | N.S. | N.S. | N.S. | N.S. | N.S. |
| TMR≥0.04 ($x_{TMR}$=1) | - | - | - | 2.666 (1.272-5.588) | 0.980 | 0.009 | 2.616 (1.239-5.522) | 0.962 | 0.012 |

| LVEF>35% | **Clinical Multivariable** | | | **ECG Multivariable** | | | **Combined Multivariable** | | |
| --- | --- | --- | --- | --- | --- | --- | --- | --- | --- |
|  | HR (95% CI) | β | *p* | HR (95% CI) | β | *p* | HR (95% CI) | β | *p* |
| CIA | - | - | - | 3.915 (1.312-11.684) | 1.365 | 0.014 | 3.915 (1.312-11.684) | 1.365 | 0.014 |
| Δα^Tpe^≥0.028 ($x_{{\Delta\alpha}_{Tpe}^{SCD}}$=1) | - | - | - | N.S. | N.S. | N.S. | N.S. | N.S. | N.S. |
| Δα^QT^≥0.228 ($x_{{\Delta\alpha}_{QT}^{SCD}}$=1) | - | - | - | 4.630 (1.512-14.179) | 1.533 | 0.007 | 4.630 (1.512-14.179) | 1.533 | 0.007 |

HR = Hazard ratio; NYHA = New York Heart Association; LVEF = Left Ventricular Ejection Fraction; NSVT = Non-Sustained Ventricular Tachycardia; VPB = Ventricular Premature Beat; IAA = Index of Average Alternans; TS = Turbulence Slope; TMR = T-wave Morphology Restitution
