# Supplementary material for: Sudden cardiac death and pump failure death prediction in chronic heart failure by combining ECG and clinical markers in an integrated risk model
Source: PLoS One. 2017 Oct 11;12(10):e0186152. doi: 10.1371/journal.pone.0186152 (PMC5636125; doi:10.1371/journal.pone.0186152)
Supplement: S5 Table — (DOCX) [file pone.0186152.s007.docx]

**S5 Table: Multivariable predictors of PFD in both reduced and preserved LVEF populations.**

| LVEF≤35% | **Clinical Multivariable** | | | **ECG Multivariable** | | | **Combined Multivariable** | | |
| --- | --- | --- | --- | --- | --- | --- | --- | --- | --- |
|  | HR (95% CI) | β | *p* | HR (95% CI) | β | *p* | HR (95% CI) | β | *p* |
| Diabetes ($x_{Diab}$=1) | 2.713 (1.442-5.102) | 0.998 | 0.002 | - | - | - | 2.437 (1.285-4.623) | 0.891 | 0.006 |
| NYHA class III ($x_{NYHA}$=1) | 3.253 (1.716-6.168) | 1.180 | <0.001 | - | - | - | 2.644 (1.392-5.022) | 0.972 | 0.0033 |
| ARB or ACE inhibitors ($x_{Inh}$=1) | 0.256 (0.105-0.623) | -1.364 | 0.003 | - | - | - | 0.249 (0.102-0.610) | -1.391 | 0.002 |
| Beta-blockers ($x_{\beta}$=1) | N.S. | N.S. | N.S. | - | - | - | N.S. | N.S. | N.S. |
| Maximum HR [per 1 SD increment] | - | - | - | N.S. | N.S. | N.S. | N.S. | N.S. | N.S. |
| RR range [per 1 SD increment] | - | - | - | 0.710 (0.501-1.007) | -0.342 | 0.055 | N.S. | N.S. | N.S. |
| CIA  ($x_{CIA}$=1) | - | - | - | N.S. | N.S. | N.S. | N.S. | N.S. | N.S. |
| Δα^Tpe^≤0.022 ($x_{{\Delta\alpha}_{Tpe}^{PFD}}$=1) | - | - | - | 2.484 (1.306-4.724) | 0.910 | 0.006 | 2.565 (1.345-4.890) | 0.942 | 0.004 |
| TS≤2.5ms/RR ($x_{TS}$=1) | - | - | - | 4.192 (1.722-10.205) | 1.433 | 0.002 | 3.667 (1.511-8.899) | 1.299 | 0.0041 |

| LVEF>35% | **Univariable** | | **Clinical Multivariable** | | | **ECG Multivariable** | | | **Combined Multivariable** | | |
| --- | --- | --- | --- | --- | --- | --- | --- | --- | --- | --- | --- |
|  | HR (95% CI) | *p* | HR (95% CI) | β | *p* | HR (95% CI) | β | *p* | HR (95% CI) | β | *p* |
| **Clinical variables** | | | | | | | | | | | |
| Age [per 1 SD increment] | 2.093 (1.221-3.591) | 0.007 | 2.093 (1.221-3.591) | 0.739 | 0.007 | - | - | - | 1.736 (1.009-2.986) | 0.552 | 0.046 |
| **Holter ECG variables** | | | | | | | | | | | |
| TS≤2.5ms/RR ($x_{TS}$=1) | 3.924 (1.564-9.844) | 0.004 | - | - | - | 3.924 (1.564-9.844) | 1.367 | 0.004 | 2.918 (1.118-7.618) | 1.0711.07 | 0.029 |

CIA = complex index of arrhythmia; HR = Hazard ratio; SD = Standard Deviation; NYHA = New York Heart Association; ARB: Angiotensin Receptor Blocker; ACE: Angiotensin-Converting Enzyme; LVEF = Left Ventricular Ejection Fraction; TS = Turbulence Slope
